# Optimizing peer review rounds in radiation oncology: a scoping review

**DOI:** 10.3389/fonc.2026.1686796

**Published:** 2026-02-06

**Authors:** Jessica Zhang, Conrad Bayley, Marcus Vaska, Sangjune Laurence Lee

**Affiliations:** 1 Department of Medicine, University of Alberta, Edmonton, AB, Canada; 2 Department of Oncology, University of Calgary, Calgary, AB, Canada; 3 Department of Radiation Oncology, Arthur J.E. Child Comprehensive Cancer Center, Calgary, AB, Canada; 4 Library Services, Acute Care Alberta, Edmonton, AB, Canada

**Keywords:** chart review, efficiency, health care, peer review, radiation oncology

## Abstract

**Purpose:**

In radiation oncology (RO), peer review (PR) rounds are essential for ensuring quality care, enhancing team communication, and identifying areas for improvement in radiotherapy (RT) plans. However, time constraints, lengthy discussions, and imbalanced team contributions often hinder effective PR. This scoping review examined novel tools and processes to enhance PR efficiency and experience in modern academic centers.

**Materials and methods:**

We queried six databases [MEDLINE (Ovid), EMBASE, PubMed, Cochrane Library, CINAHL, and MEDLINE (Ebsco)] and the gray literature, yielding 8,955 citations. Studies were excluded if they (1) were focused on comparisons involving paper-based rounds, (2) lacked clear relevance to PR processes in RT, or (3) did not explicitly address efficiency within PR activities.

**Results:**

Twelve studies focusing on PR structure and efficiency-related processes were included. Of the identified, 11/12 explored various structural formats to improve facilitation, 5/12 discussed automated tools, and 2/12 evaluated checklists. Only half of studies reported a PR-associated time burden, with 2/12 reporting positive post-implementation changes. The remaining studies did not measure comparative times.

**Conclusions:**

This scoping review reveals the lack of work on innovative approaches to optimize PR rounds in RO, despite the commonly reported participation barrier of high time commitment. Our findings highlight the importance of integrating automation in order to streamline facilitation methods and tools such as checklists to reduce inefficiency, given PR’s essential role in patient safety and clinical learning. Future research should prioritize the development and evaluation of time-saving strategies and tools for PR in RO workflow to optimize its sustainability and impact.

## 1 Introduction

In radiation oncology (RO), peer review (PR) rounds—also referred to colloquially as quality assurance (QA) rounds—are conducted routinely to ensure that standards of care are met, to foster communication within the multidisciplinary team, and to identify potential deficiencies or areas for improvement (1–4). PR has long been a pillar of Canadian radiotherapy (RT) standards, promoting the delivery of safe and high-quality care (1, 5). Canadian initiatives, such as Ontario’s Radiation Treatment Program Implementation Plan for 2019–2023, identified PR as a priority for enhancing the quality and safety of RT across cancer centers. They championed the evaluation of “best practices” in PR, the incorporation of novel strategies, and utility of artificial intelligence (AI) (6). Despite its recognized importance, several barriers to effective QA rounds have been reported, including scheduling constraints, lengthy discussion periods exceeding allotted time, and inconsistent participation from members of the multidisciplinary team (4, 7, 8).

The impact of PR on treatment planning and clinical outcomes has been an ongoing area of investigation. A 2013 survey conducted by the American Society for Radiation Oncology (ASTRO) found that over 90% of respondents had made changes to treatment plans as a result of PR at some point in their career. However, the estimated overall impact on case revisions at that time was 4%–7% of those brought forth to rounds (9). More recent evidence from a systematic review and meta-analysis from 2025 presents a contrasting view, which reported that PR influenced treatment plan changes in over 25% of cases (10). In specialized contexts, namely, the use of magnetic resonance linear accelerators (MR-Linacs), PR can lead to modifications to 36.4% of treatment plans (11). For delineation of planning target volumes (PTVs) and organs at risk (OARs), recommended changes were observed in approximately 10% of 7,645 reviewed cases (12).

From the perspective of radiation oncologists, multiple surveys have assessed experiences with PR. Canadian radiation oncologists generally recognize the value of PR in improving treatment planning; however, time constraints and lack of protected time for participation are consistently identified as major barriers (7, 8). Notably, implementation of protected time policies has been associated with improved physician attendance (13). These findings suggest that strategies to reduce time burden and improve workflow efficiency could enhance engagement and the overall effectiveness of PR. The present study seeks to answer the question: for the RO workflow within academic RT centers, what effect do novel tools and strategies have on the PR process in terms of decreased time burden, ability to correct treatment plans, and general implementation as compared to the current standard QA process?

## 2 Methods

This review follows the 2018 PRISMA extension for scoping reviews (PRISMA-ScR) protocol (14). In collaboration with a health sciences librarian (M.V.), a comprehensive search strategy was developed to identify studies addressing efficiency in PR rounds within the context of RT (see the **Supplementary Materials**). In August 2024, systematic searches were conducted across six electronic databases: MEDLINE (Ovid), EMBASE, PubMed, Cochrane Library, CINAHL and MEDLINE (Ebsco). Gray literature sources were also included. The search yielded a total of 8,955 citations. Studies were excluded if they (1) focused on comparisons involving paper-based rounds, (2) lacked clear relevance to PR processes in RT, or (3) did not explicitly address efficiency within PR activities. Title and abstract screening, followed by full-text review, were independently performed by two reviewers (C.B. and J.Z.) using Covidence (Veritas Health Innovation, Melbourne, Australia, 2024). Discrepancies were resolved through consensus. Data extraction was conducted using a standardized template to ensure consistency across included studies (see the **Supplementary Materials**).

A reflexive thematic analysis aligned with Braun and Clarke’s framework was performed to synthesize findings (15). PR approaches were thematically grouped into three domains: automation, facilitation, and the use of checklists. For the purpose of this review, automation refers to the integration of tools or software that partially or fully automate elements of the PR process, while facilitation relates to organizational strategies and session structure. Checklists represent a standardization tool aimed at improving consistency and completeness of plan evaluation.

## 3 Results

**Figure 1:**
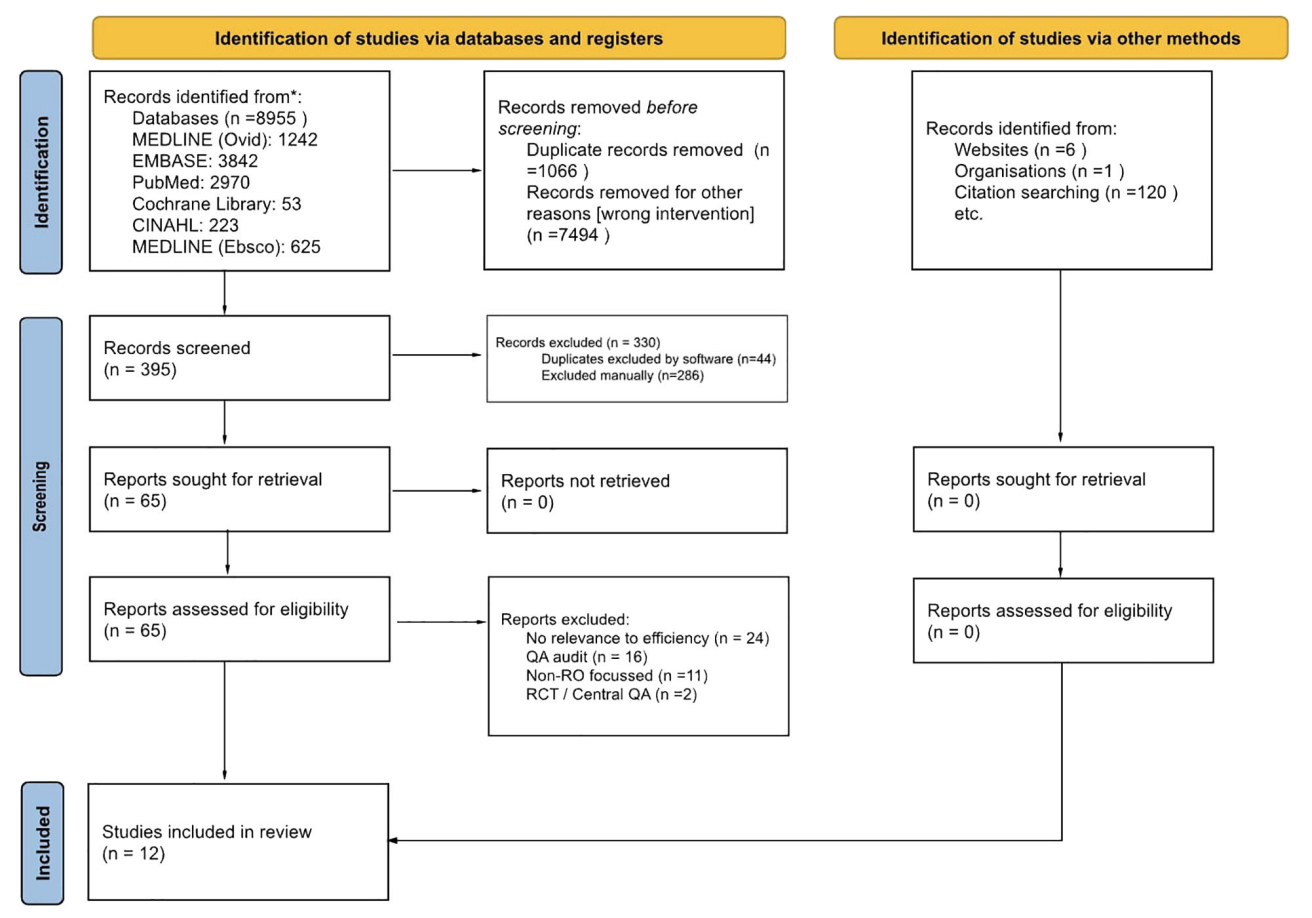
PRISMA 2020 flow diagram for radiation oncology peer review scoping review*. *Multiple overlapping studies identified from systematic search and citation searching. Source: Page MJ, et al. *BMJ* 2021;372:n71. doi: 10.1136/bmj.n71.

A total of 395 studies were independently screened by title and abstract, with 65 selected for full-text review (**Figure 1**). Of these, 12 studies met inclusion criteria, focusing specifically on the structure and efficiency of PR processes in RT.

Among the included studies, 11 out of 12 (92%) examined modifications to PR structure aimed at improving facilitation. Five studies (40%) evaluated the use of automated tools, while two (17%) discussed structured checklists. These themes are summarized in **Table 1**. Only six studies (50%) reported data on time burden associated with PR processes, and of these, only two studies described measurable improvements in efficiency following implementation of new interventions, discussed below. The remaining studies did not report comparative time metrics. The study aims, interventions, methods, results, and limitations are summarized in **Table 2**.

**Table 1:** Summary of thematic analysis.

Major themes	Definition	Included studies
Automation	Integration of tools or software that partially or fully automate elements of the PR process	5
Checklists	Standardization tool aimed at improving consistency and completeness of plan evaluation	2
Facilitation	Organizational strategies and session structure	10

**Table 2:** Overview of included studies on peer review efficiency in radiation oncology.

First author [country] study title	Aim	Type	Methods and results	Time metrics	Limitations per author
Kim 2023 [CAN/KOR] Effect of Radiation Therapy Quality Assurance on Nasopharyngeal Carcinoma: Usage of a Novel, Web-Based Quality Assurance Application	Evaluate RT volumes using web-based tool	Automation	Retrospective analysis of 332 NPC plans. Tool identified coverage errors linked to patient outcomes.	N	Single center, retrospective, small event numbers.
Ali 2024 [US] Implementation of a Novel Chart Rounds (NCR) Application to Facilitate Peer Review in a Virtual Academic Environment	Improve PR via automation + reformatting sessions	Automation facilitation	NCR was 6 site-based 1-h sessions that reviewed 1,160 plans; feedback integrated into workflow. Able to identify areas of improvement.	Y	Scoring cards added time; delayed completion may affect plans.
Talcott 2024 [US] The Future of Safety and Quality in Radiation Oncology	Commentary on future best practices	Automation facilitation	Endorses prospective site-specific PR, AI-generated scorecards (e.g., ClearCheck) for efficiency.	N	Not specified.
Vijayakumar 2019 [US] Chasing Zero Harm in Radiation Oncology: Using Pre-treatment Peer Review	Report on group consensus peer review	Automation facilitation	4-step PR with automated DVH tool and open voting. Minimize clinical hierarchy.	N	Time investment; requires multidisciplinary adherence.
Albert 2018 [US] Analysis of a Real Time Group Consensus Peer Review Process in Radiation Oncology: An Evaluation of Effectiveness and Feasibility	Quantify plan changes and resource needs in PR	Automation facilitation	Group consensus based DVH scorecards; avg. 8 min/case.	Y	Not specified.
Fong 2017 [UK] Implementing Head and Neck Contouring Peer Review without Pathway Delay: The On-demand Approach	Compare on-demand vs. weekly PR	Facilitation	On-demand PR faster (mean 19.7 h vs. 27.9 h); fewer treatment delays; dosimetrists received avg 2 extra days per case.	Y	Small sample (*n* = 62 cases); possible volunteer bias.
Ludmir 2024 [US] Implementation and Efficacy of a Large-Scale Radiation Oncology Case-Based Peer-Review Quality Program across a Multinational Cancer Network	5-year evaluation of prospective PR across 8 centers	Facilitation	Weekly site-based virtual PR. Non-concordance rates decreased over time.	N	Accuracy of centers’ own PR; Lack of focused physics review; No measure of delayed treatment start.
Surucu 2019 [US] The Impact of Transitioning to Prospective Contouring and Planning Rounds as Peer Review	Assess implementation of daily CPR rounds	Facilitation	Added CPR rounds; maintained avg 5-day CT-to-treatment. 6–8 min/case, no added delay.	Y	Not specified.
Bhattacharyya 2024 [India] Enhancing Quality Assurance in Radiotherapy for Gynaecological Cancers: Implementation of an On-Demand Peer Review Process	Evaluate on-demand PR	Facilitation	Prospective evaluation of PR outcomes. On-demand system timelier.	N	Single site; no formal comparison to weekly PR.
Gulstene 2024 [CAN] Evaluating Peer Review of Palliative Radiation Plans at a Canadian Tertiary Care Cancer Center	Retrospective review of 1:1 (independent) vs. team PR	Facilitation	Independent PR halved physician time (9.7 min vs. 20.5 min); similar feedback rates.	Y	Unclear ideal PR rate; unable to truly compare efficacy.
Lewis 2021 [US, Belgium, UK] Structure and Processes of Existing Practice in Radiotherapy Peer Review: A Systematic Review of the Literature	Systematic review of best PR practices	Facilitation Checklist	17 studies included. Recommends weekly, site-based ≥1 h PR with 8–10 cases reviewed and standard data reporting.	Y	Not specified.
Swaminath 2020 [CAN] Development of Best Practices of Peer Review for Lung Radiation Therapy	Determine best PR practices for lung	Checklist	National Delphi-based consensus; compiled 6 core elements.	N	Unable to capture every RO/practice style.

### 3.1 Automation

Over the past decade, the application of automation within PR has gained traction, with an emphasis on standardizing inherently qualitative and subjective processes (16). There has been more recent exploration of the potential role of AI in consolidating PR activities. Talcott et al. (17), for instance, generally described the use of AI to generate “score cards” and support decision-making in QA rounds (17). However, concerns related to automation bias, user complacency, and the risk of systematic errors remain notable. Several innovations have been described, including automated tools designed to assess case complexity, generate PR case lists with relevant clinical information, and produce dose volume histogram (DVH) analyses (18–20). Barry et al. (18) developed a simple automated scoring system based on complexity and planning for breast RT that was easily integrated into PR (18). Ali et al. (19) implemented a similar standardized scoring system that was able to integrate feedback directly into physician feedback (19). Albert et al. (20) employed a color-based scorecard if dose constraints were met or not, signified by green and red, respectively. They reported a mean presentation time of 8 min per case (20). Furthermore, Kim et al. (16) reported the ability of novel cloud-based platform to rapidly evaluate target volume delineation in head and neck RT against 2018 international standards. They reported an undercoverage of the cavernous sinus but otherwise had high compliance rates (16). The studies on automation mentioned did not otherwise elaborate on improvements to efficiency. These approaches demonstrate the growing interest in leveraging automation to streamline PR and improve standardization.

### 3.2 Facilitation

Modifications to PR round structure have also been explored as a means of enhancing efficiency. Decreasing the number of participants needed to successfully run a PR session is one possible solution. Ali et al. (19) found that when they implemented a New Chart Review (NCR) structure consisting of six 1-h site-specific sessions per week, it allowed improved review of palliative plans and reduced absolute participant number at each round when compared to Standard Chart Review (SCR), consisting of two 90-min weekly sessions encompassing all treatment sites (19).

An emerging model, “on-demand” PR, was designed to prioritize cases requiring urgent review while avoiding unnecessary treatment delays. Fong et al. (21) conducted a pilot project where four head and neck radiation oncologists were able to voluntarily submit cases for PR “on-demand”. They then compared the time taken for this process to their traditional weekly review approach. The authors observed a statistically significant reduction in time burden (*p* < 0.0001) with this model, though there were challenges related to voluntary participation and uneven contribution among radiation oncologists. Furthermore, they noted that there is no current agreement on priority classification for cases. The authors recommended supplementing on-demand reviews with periodic consensus meetings to maintain educational opportunities and ensure an equitable workload distribution (21). A subsequent study also reported favorable perceptions of on-demand PR in improving timeliness (22).

Group consensus-based PR remains a widely adopted approach for developing tools or evaluating aspects of PR (20, 23, 24, 35). A recent study by Gulstene et al. (25) suggests that independent PR may also be a viable alternative. They reported a reduction of approximately 50% in physician time burden (20.5 ± 6.0 vs. 9.7 ± 6.4 min) per plan compared to the traditional team approach. There were otherwise no significant differences in the rate of peer feedback, plan modifications, or the relationship between RO attendance and likelihood of plan changes between the two structures (29).

Prospective PR, conducted prior to treatment initiation, continues to be favored over retrospective models, as it enables early identification and correction of planning issues (26, 30). For example, Surucu et al. (27) integrated daily contouring and planning rounds (CPRs) alongside weekly chart rounds, facilitating the presentation of six to seven cases within a 45-min session. Importantly, the addition of daily CPR did not increase the overall time from simulation to treatment start, demonstrating the feasibility of prospective approaches without affecting efficiency (31).

### 3.3 Checklists

The use of medical checklists has been well-established in improving safety and standardization across clinical disciplines. National surveys within American RO have demonstrated strong interest in checklist adoption (32). Two studies described the development of consensus-based checklists for PR of RT treatment plans (28, 33). Swaminath et al. (2020) used a modified Delphi process involving a preliminary steering committee with subsequent pan-Canadian panel to generate a best practices guideline for curative lung RT. They reported high consensus that a second RO should carry out PR. Across locally advanced (LA) and SABR (stereotactic ablative body RT) cases, both shared elements of RT indications, gross tumor volume (GTV), clinical target volume (CTV), internal target volume (ITV), and dose/fractionation. LA also included normal lung dosimetry, while SABR had composite plan review deemed as essential (28). Boyd et al. (29) employed the nominal group technique (NGT) with a panel of 10 American academic radiation oncologists to create the first consensus external beam treatment plan checklist for generalized application. Their checklist included patient identification, imaging modality, treatment intent and modality, clinical trial participation, intended target volumes, OARs, presence of devices, beam energy, position, 3D dose distribution, and DVH (33). Between these two studies, their respective elements were overall similar. These tools were designed for comprehensive review and consistency in documentation and feedback.

## 4 Discussion

Benefits of PR include improving RT planning and delivery to maintain the highest standard of patient care. Undergoing the PR process also promotes the development of new RT protocols, providing an interdisciplinary learning environment to enhance team-based collaboration both within RT and across oncologic specialties (30).

Although efficiency remains a less explored area in the landscape of PR, other changes to the process have been associated with positive outcomes. For example, grading systems, whether manual or automated, have become more common. A 10-year prospective evaluation involving 20,069 cases implemented a grading system to categorize review outcomes: “A” (no changes), “B” (minor changes), and “C” (major changes). Over time, the proportion of “A” scores declined, while “B” scores increased, suggesting increased scrutiny and reduced incidence of major errors (“C” scores) in treatment planning (31). These findings imply improvements in PR processes and/or the adoption of more rigorous planning standards. In the last decade, there has also been a notable shift toward virtual PR platforms, aimed at enhancing accessibility regardless of institution size or geographic location, and improving equity in care delivery when PR is made accessible to all centers and patients (32). Of note, there remains continued support for prospective PR models, which allow for modifications to be implemented prior to treatment initiation (33, 34).

Among the literature reviewed, only one formal systematic review specifically addressed existing PR practices in RT. This review proposed recommendations for the structural components of PR rounds and advocated for the consistent documentation of outcomes using standardized criteria. Notably, none of the included studies reported time metrics or overall efficiency associated with their PR processes (35). The evaluation of efficacy in RO PR rounds is not a novel concern. As early as 1999, studies identified a lack of formal analysis linking PR structure to quality outcomes in RT QA (36). The present review reaffirms this gap: despite increasing awareness of the time-intensive nature of PR rounds, few studies have specifically addressed strategies to improve their efficiency. This deficiency is particularly notable given that time burden remains one of the most frequently cited barriers to consistent PR participation.

Inherent limitations of the PR process include its resource-intensive nature alongside variations in frequency, case selection, and structure. Increasing automation in the PR process may help with standardization, at the risk of introducing automation bias, complacency, and systematic error if we begin to rely on tools for guidance. To prevent this over-reliance, it is important to maintain transparent documentation and a clear understanding of tools’ limitations, so that PR remains human-driven rather than machine-replaced. Critical thinking remains central to fulfilling the ultimate goal of patient safety by conducting PR rounds, and so automation should help improve efficiency and consistency without adversely impacting clinical judgment. Other unanswered questions remain such as the associated cost with PR processes and how much patients benefit from the changes made (13, 32). While there is robust support for the impact of PR on plan quality and error reduction, there is a lack of direct, prospective evidence establishing a relationship between PR rounds and improved patient-level outcomes including local control, survival, or toxicity. However, given the importance of safety checks and plan quality, it remains widely supported that PR is a critical process for optimizing patient care across multiple specialties. In a 2017 systematic review, Brunskill et al. found that PR led to changes in clinical planning for 11% of included cases, a similar rate to adjacent specialties such as pathology and radiology (37).

## 5 Limitations

This scoping review ultimately identified a small number of relevant studies, which may reflect limitations in the search strategy to have picked up all targeted interventions. The inclusion criteria were set to English language only, which may have also limited the search results. For quality analysis of studies, we commented on limitations as reported by the authors but did not conduct formal independent analyses.

## 6 Future directions

Despite ongoing variability in PR implementation across academic centers, a general consensus exists regarding its timing and core components. However, critical questions remain, particularly concerning cost-effectiveness and the tangible clinical benefits for patients (13, 32). Emerging areas of exploration include determining the optimal number of physicians required for team-based PR and assessing the utility of AI-driven tools to detect contouring or planning errors (25, 38–40). With advancing capabilities in image recognition and automated QA, AI-based platforms may offer valuable support to enhance accuracy and consistency (16). As a notable example, Talcott et al. (41) introduced deliberate errors into PR rounds and observed a detection rate of 55%, with earlier-presented cases demonstrating higher detection likelihood—highlighting the potential benefits of shorter or more focused review sessions (41). The consistent reporting and evaluation of time spent in PR, as advocated by Albert et al. (20), could also provide valuable benchmarks for institutional improvement (20).

As Hendee and Herman (2011) astutely put, “A single error that harms a radiation therapy patient is one error too many” (42). While technological advancements continue to evolve, the role of integrated human oversight remains indispensable. Moving forward, the development of tools and workflows must be grounded in the shared objective of delivering safe, effective, and patient-centered care. PR processes should not only be rigorous but also be feasible—balancing efficacy with efficiency to best support both clinical teams and the patients they serve.

## 7 Conclusion

This scoping review highlights the need to optimize the efficiency and structure of PR in RT through diverse strategies such as automation, session formatting, and standardized tools including checklists. While more novel approaches, such as on-demand review and AI-supported tools, demonstrate promising outcomes, evidence remains limited with respect to direct comparisons of time efficiency and ultimate clinical impact. As PR remains integral to clinical decision-making, patient safety, and professional development in RO, further innovation is essential to ensure its sustainability and value in modern practice. More studies that aim to quantify time burden, conduct objective comparisons of software solutions, and evaluate clinical outcomes from changes made during the PR process are warranted to evaluate the long-term effectiveness, feasibility, and scalability of these interventions across varied clinical settings.

## Data Availability

The original contributions presented in the study are included in the article/**Supplementary Material**. Further inquiries can be directed to the corresponding author.
