# Supplementary material for: Optimizing peer review rounds in radiation oncology: a scoping review
Source: Front Oncol. 2026 Feb 6;16:1686796. doi: 10.3389/fonc.2026.1686796 (PMC12920248; doi:10.3389/fonc.2026.1686796)
Supplement: Supplementary file 1 [file Table1.docx]

Supplementary Material

# Search Strategy

| **Concept** | **Synonym** |
| --- | --- |
| Quality Assurance/Chart Rounds | “quality assurance” [Keyword]; quality assurance, health care [MeSH]; “chart round*” [Keyword] |
| Radiation Oncology | “radiation oncology” [Keyword, MeSH] |
| Peer Review | “peer review” [Keyword, MeSH] |

- Suggested search string: (“quality assurance” OR “chart round*”) AND “radiation oncology” AND “peer review”
- Databases: MEDLINE (Ovid); EMBASE; PubMed; Cochrane Library; CINAHL; MEDLINE (Ebsco)
- Grey Literature: AHS Insite; Open Archives Initiative (OAI)Ster; [ClinicalTrials.gov](http://clinicaltrials.gov); American Society of Clinical Oncology (ASCO), European Society for Medical Oncology (ESMO); Google; Google Scholar; B.C. Cancer Agency; Cancer Care Ontario
- Limits: language (English), publication date (1995—present)

1. **Extraction Template Elements**

- General: study ID, title, year of publication, lead author, country/countries of study
- Type: automation, facilitation, checklist (multi-select)
- Study design: quality improvement, commentary, both
- Methods
- Results
- Takeaways (to be used in discussion)
- Limitations per authors
- Study funding sources
- Possible conflicts of interests per study authors
